# A Combination of Cabozantinib and Radiation Does Not Lead to an Improved Growth Control of Tumors in a Preclinical 4T1 Breast Cancer Model

**DOI:** 10.3389/fonc.2021.788182

**Published:** 2021-12-08

**Authors:** Norman Reppingen, Alexander Helm, Laura Doleschal, Marco Durante, Claudia Fournier

**Affiliations:** ^1^ Department of Biophysics, GSI Helmholtz Center for Heavy Ion Research, Darmstadt, Germany; ^2^ Department of Biology, Technische Universität Darmstadt, Darmstadt, Germany; ^3^ Department of Condensed Matter Physics, Technische Universität Darmstadt, Darmstadt, Germany

**Keywords:** Cabozantinib, radiotherapy, SBRT, triple-negative breast cancer, 4T1 cells

## Abstract

The tyrosine kinase inhibitor Cabozantinib has been applied in clinical studies in combination with radiotherapy. We investigated the effect of such combination on triple-negative 4T1 cells as a metastatic breast cancer model *in vitro* and *in vivo* upon inoculation in BALB/c mice. *In vitro* assays indicated a potential for improved effects using the combination. Both Cabozantinib (2.5 µM) and 10 Gy of 250 kV x-rays were able to cease the growth of 4T1 cells as revealed by growth curves. In a clonogenic survival assay, the effect of Cabozantinib added on the effects of irradiation and the effectiveness of inhibiting the clonogenic survival was found to be 2 (RBE_10_). Additionally, cell death measurements of apoptosis plus necrosis revealed a synergistic effect when combining irradiation with Cabozantinib. Surprisingly, however, *in vivo* tumor growth kinetics showed no additional effect in growth control when irradiation was used together with Cabozantinib. Since both ionizing radiation and Cabozantinib are acknowledged to feature immunogenic effects, we additionally investigated the effect of the treatments on lung metastases. No difference to the control groups was found here, neither for irradiation nor Cabozantinib alone nor in combination. Yet, upon analysis of the mice’ livers, CD11b-positive cells, indicating immune suppressive myeloid derived suppressor cells were found diminished following treatment with Cabozantinib. In conclusion, despite promising *in vitro* controls of the combination of Cabozantinib and irradiation, tumor growth control was not increased by the combination, which was true also for the occurrence of lung metastases.

## Introduction

The combination of treatments including immunotherapy (1), small molecule pharmacology (2, 3) and radiotherapy (4, 5) is a rapidly growing and promising field. Radiotherapy (RT) may induce immunologically relevant molecular and cellular effects, including abscopal effects with shrinking or vanishing tumors outside the radiation field (6) and is hence a match for combination therapies featuring immune responses. Cabozantinib, a tyrosine kinase inhibitor, was shown to optimize anti tumor immunity, reducing the prevalence of regulatory T cells (7) and myeloid-derived suppressor cells (MDSCs) in mouse models (7, 8). Clinical trials combining both have been set up for treatment of glioblastoma (in combination with temozolomide) (9) or sarcomas of the extremities (NCT04220229).

Triple-negative breast cancer (TNBC) represents a challenging therapeutic target due to the highly invasive nature and relatively low response to therapeutics. TNBC is managed with conventional therapeutics, including RT, often leading to systemic relapse since there is an absence of specific treatment strategies for this tumor subgroup (10). In a Phase II study in TNBC patients, Cabozantinib increased the number of circulating CD8+ T-Cells and decreased the presence of CD14+ myeloid cells (11). RT administered in stereotactic body radiation therapy (SBRT) character is held to trigger a powerful immune activation (12). Combination of RT in a SBRT-like character and Cabozantinib may be beneficial in the treatment of TNBC. We investigated the potential of such a combined therapy using the murine 4T1 model of metastatic breast cancer.

Both Cabozantinib (2.5 µM) and 10 Gy of 250 kV x-rays were able to cease the growth of 4T1 cells *in vitro* as measured by reduced growth and reduced clonogenic survival. We observed that the effect of Cabozantinib added on the effects of irradiation. Additionally, cell death measurements revealed a slightly synergistic effect. In contrast, *in vivo* tumor growth kinetics showed, compared to Cabozantinib treatment alone, no additional effect in growth control for combined treatment. With respect to the occurrence of lung metastases, no difference to the untreated control groups was found here. Yet, upon analysis of the mice’ livers, CD11b-positive cells, indicating immune suppressive myeloid derived suppressor cells (MDSC) were found diminished following treatment with Cabozantinib. In conclusion, despite promising *in vitro* results of the combination of Cabozantinib and irradiation, tumor growth controls was not increased by the combination, which was true also for the occurrence of lung metastases.

## Material and Methods

### Animal and Cell Models

4T1 cells were from ATCC and maintained in RPMI culture medium from Merck with 9.6% of fetal bovine serum (Biochrom AG) and 1% penicillin/streptomycin solution from Invitrogen. For *in vitro* experiments, Cabozantinib was used at 2.5 µM as in Kwilas et al. (7) in the supplemented medium. Irradiation of cells was furnished with 250 kV x-rays from the IV320-13 from Seifert using a filter consisting of 7 mm Be, 1 mm Cu and 1 mm Al, using a SN4 dosimeter from PTW at a dose rate of 2.5 Gy/min.

BALB/c mice were kept in accordance with federal policies on animal research. The corresponding ethical approval code is 23 177-07/G 15-8-058. After a week of adapation, 1*10^5^ 4T1 cells in 20 µl PBS were injected into the second mammary fat pad under ketamine/xylazine narcosis. Tumor size was measured every three days using vernier calipers. Tumor volume was calculated using the formula V = (A*B^2^)/2, with A as the largest and B as the smallest diameter of the tumor. Mice were sacrificed 26 days post tumor inoculation, corresponding to 12 days after radiation exposure. For the count of lung metastases, the lungs were infused with blue ink upon resection, counting the superficially visible metastases.

Irradiation was performed with a 320 kV x-ray tube from X-RAD at a dose rate of 0.47 Gy/min at day 14 after tumor cell inoculation, using lead collimation to shield non-tumor areas under ketamine/xylazine narcosis, which was also applied to the unexposed groups. Drugging with Cabozantinib started on day 5 after tumor inoculation and lasted 21 days, until the end of the study. The drug was applied *via* mouse chow as a purified ingredient diet prepared by Research Diets, New Brunswick, NJ, containing 66.7 mg/kg Cabozantinib malate salt (LC Labs), which was established to correspond to a daily dose of 10 mg/kg (7). An untreated control group did not receive neither irradiation nor Cabozantinib. The number if mice assigned to the experimental groups were 8 (negative controls and 14 Gy) and 12 (Cabozantinib and 14 Gy + Cabozantinib), respectively.

### Masson Goldner Staining and CD11b Staining

The liver samples were fixed in Histofix (Carl Roth) and stored in 70% Ethanol, transferred into paraffin blocks *via* ethanol/xylene stages, and cut with a rotary microtome from Leica in slices of about 5 µm. After deparaffination in xylene and rehydration, the samples were stained according to the method of Masson and Goldner. For immunohistological staining of CD11b, following deparaffination and antigen retrieval in citrate buffer in a household microwave oven for 20 minutes and washing in PBS buffer, the samples were blocked with goat serum (Sigma) diluted in PBS, supplemented with 1% of Triton X-100 for 30 minutes. The rabbit primary antibody directed against CD11b (abcam 133357, clone EPR1344), was applied at a concentration of 1:4000 in PBS and incubated over night at 4°C in the dark. After washing with PBS, the slides were incubated with blocking reagent for 30 min. followed by 2 h of incubation with a HRP-conjugated anti rabbit secondary antibody (abcam 6721, polyclonal) at a dilution of 1:100 (also in PBS). The staining was developed using the ImmPACT VIP substrate according to manufacturer specifications, dehydrated over alcohol and xylene stages and embedded in Eukitt (Sigma). Samples were captured using a BX61 microsope from Olympus equipped with plan - apochromatically corrected lenses.

### Assessment of Cell Growth

Petri dishes were seeded with 1*10^4^ 4T1 cells one day prior to treatment with irradiation and/or Cabozantinib as described above. Cells were harvested and counted 24, 48, 72 and 96h following treatment using an automated cell counter (Coulter).

### Assessment of Clonogenic Survival

Clonogenic cell survival was assessed using the standard colony forming assay as described elsewhere (13). Briefly, directly after exposure cells were trypsinized, counted and plated in triplicate into 25 cm^2^ tissue culture flasks. The numbers of cells seeded were estimated to result in a statistically significant formation of at least 100 colonies. After seven days of incubation, cells were fixed and stained with a methylene blue solution. Cell clusters consisting of at least 50 cells were counted as a colony.

### Assessment of Cell Death

In order to assess cell death, the supernatant of cells was collected by decanting in 15 ml falcon tubes. The cells remaining in the T25 culture flask were washed with PBS, and the PBS added to the supernatant in the tube. 2 ml of Accumax cell dissociation solution from PAN-Biotech was added and the T25 flask incubated for 10 min. at 37 C. The liquid in the Falcon of the respective tube was unified with the Accumax solution and centrifuged at 300 g. The remaining pellet was stained using 100 µl of a staining solution consisting of propidium iodide (1 µg/ml), and Annexin-V Pacific Blue (ThermoFisher) according to manufacturer instructions (5 µl per sample) in a Buffer supplemented with Ca2+. After incubation for 20 min. at 4 C in the dark, 700 µl staining buffer was added, following by centrifugation for 5 minutes at 300 g. Using 300 µl of staining buffer, the pellet was transferred to 5 ml polystyrene tubes and subjected to flow cytometry analysis with a FACSCanto II instrument. The data was analyzed using FlowJo Software, version 10.5.3.

### Gene Expression Analysis

At least 1.5 Million cells growing in T75 culture flasks were deprived of medium and treated with 1 ml of TRIZol (Thermofisher). Further workup occurred as per manufacturer description, using 400 µl of chloroform (Alfa Aesar, Molecular Biology Reagent, stabilized with 50 ppm amylene), Iso-Propanol (Sigma, HPLC grade) and Ethanol (Roth, p.A.). The resulting RNA was characterized with a colibri-nanodrop device from Titertek Berthold and used for cDNA synthesis using the RevertAid kit (Thermofisher). For the Q-PCR runs on a StepOnePlus instrument from Applied Biosystems, 100 ng of the resulting cDNA were used per sample using vinculin as a housekeeping gene. The resulting probe volume was 25 µl, using the Quantitect primer system from QIAGEN and evaGreen 5x Q-PCR Mastermix with ROX dye from Solis Biodyne. PCR plates were from Saarstedt. The sample data was evaluated according to the Ct method with the StepOne Software version 2.3.

### Assessment of GM-CSF Release

GM-CSF release was quantified by ELISA (Thermofisher), according to manufacturer specifications, and the obtained values normalized to the cell number.

### Statistical Analysis

Data are displayed as the mean values of independent experiments plus/minus the standard error of the mean (SEM) or the standard deviation (SD) unless indicated elsewise. Significance tests were performed using GraphPad Prism version 6, GraphPad Software, La Jolla California USA.

## Results

### Cabozantinib Adds on the Effects of X-Rays in Terms of Cell Inactivation and Growth Control *In Vitro*, but This Is Not Reflected *In Vivo*

**Figure 1:**
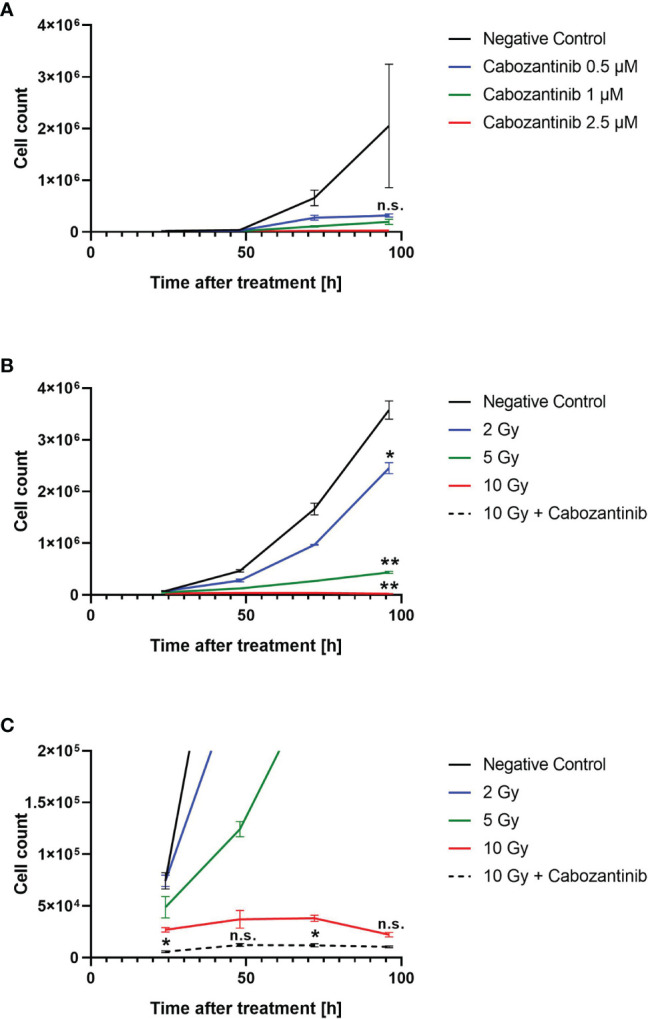
*In vitro* cell growth following treatment with Cabozantinib **(A)** or x-rays and Cabozantinib **(B)**. Cells were counted 24, 48, 72 and 96h following treatment and results are derived from experiments carried out in triplicates (mean value ± SEM). Please note that curves for 10 Gy and 10 Gy + Cabozantinib cannot be distinguished due to the scale in **(B)**. Therefore, in **(C)**, the same data as in **(B)** are displayed in a different scale. A Two-way ANOVA (at 96h, negative controls vs. each other group) revealed significant differences to the negative controls only following irradiation (**B**, *p<0.05, **p<0.01). A comparison between 10 Gy and 10 Gy + Cabozantinib revealed no significant differences at 96h **(C)**.

**Figure 2:**
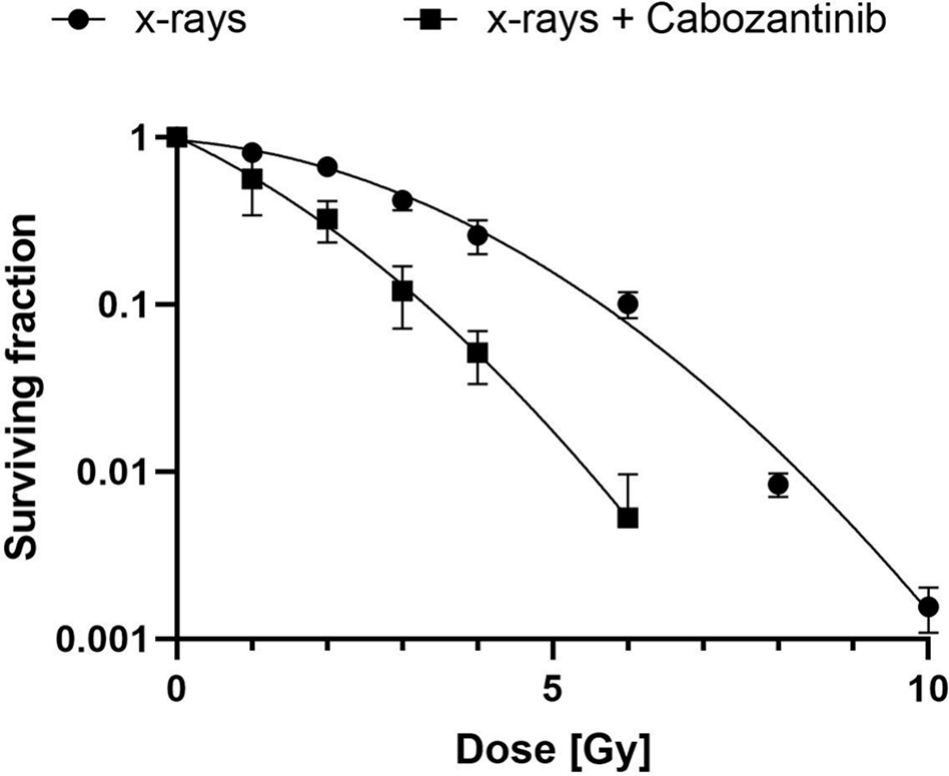
Clonogenic cell survival after exposure to x-rays and Cabozantinib. A colony formation assay was performed as described following exposure to x-rays or x-rays and 2.5 µM of Cabozantinib. Results are derived from two (x-rays + Cabozantinib) or three (x-rays) independent experiments (mean value ± SD). A t-test comparing σ-values of α revealed a significant difference (p<0.001).

**Figure 3:**
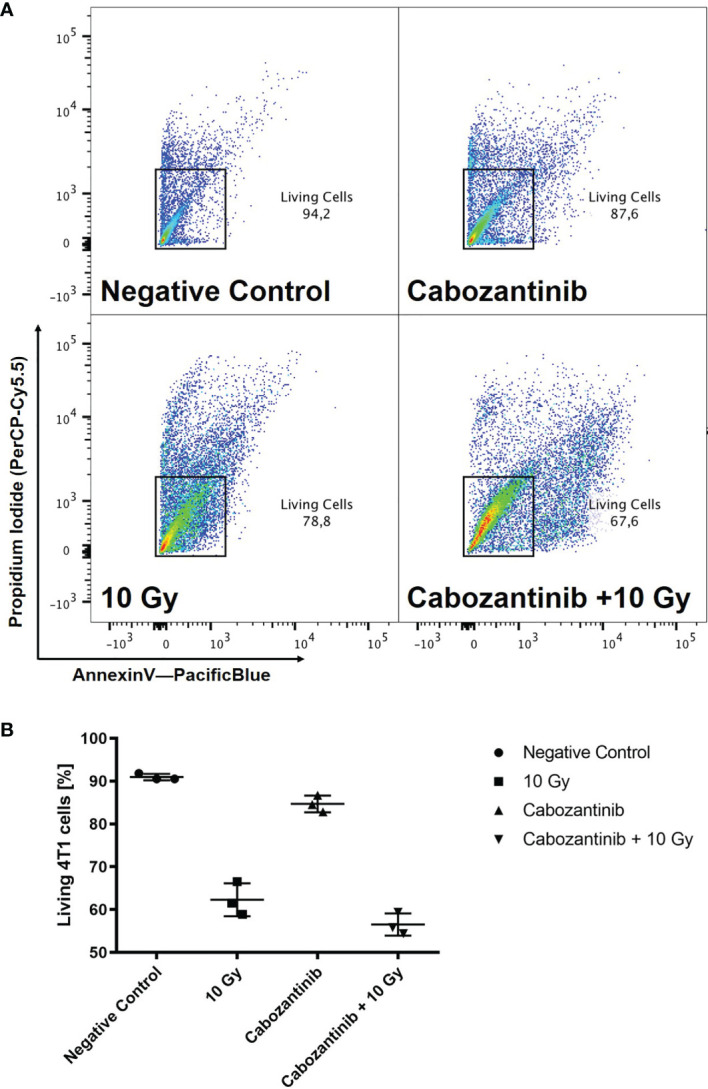
Cell death after treatment with x-rays and Cabozantinib. Cell death was assessed as described following exposure to 10 Gy of x-rays, 2.5 µM Cabozantinib or both. Exemplary scatter plots resulting from flow cytometry analysis **(A)**. Cells positive for propidium iodide and Annexin-V were excluded to obtain living cells. The example is representative for the other biological replicates. Amount of living 4T1 cells 72h after treatments **(B)**. Results are derived from three independent experiments (mean value ± SD). A One-way ANOVA revealed significant differences for 10 Gy and Cabozantinib + 10 Gy as compared to the negative control (p<0.0001), but not for Cabozantinib alone (p=0.0653) or between 10 Gy and Cabozantinib + 10 Gy (p=0.0917).

To determine the concentration of Cabozantinib and the radiation dose resulting in cell growth control, we investigated the growth of cells *in vitro* following different treatments. We tested a concentration of 2.5 µM as applied by Kwilas and colleagues (7), which resulted in control of the cell growth up to 96h after beginning of treatment, while concentrations of 0.5 or 1 µM Cabozantinib were not sufficient to do so (**Figure 1A**). As for x-rays alone, 10 Gy were found sufficient to control the growth, whereas 2 and 5 Gy failed (**Figure 1B**). Consequently, a combination of 10 Gy and 2.5 µM of Cabozantinib resulted in a total growth control up to 96h after treatment (**Figures 1B, C** **)**, which significantly differed only partly, but not at 96h. Next, we investigated clonogenic cell survival. When Cabozantinib (2.5 µM) was added, the clonogenic survival decreased and the relative biological effectiveness (RBE) was found to be about 2 at an iso-survival level of 10% (**Figure 2**). The α/β-ratios resulting from linear-quadratic fits for the curves were 1.4 (x-rays) and 7.4 (x-rays + Cabozantinib, **Supplementary Table 1**). Comparing σ values of α, the curves were found to differ significantly (p<0.001, t-test). Additionally, cell death measurements 72h after treatment revealed a significant reduction in the amount of living cells (i.e. negative both for propidium iodide and Annexin-V) following exposure to irradiation from 91.9% for negative controls to 62.3% (p<0.0001). Cabozantinib alone reduced, yet not significantly the amount of living cells to 84.7%. This was found also when Cabozantinib was added to irradiaton (56.5%, **Figure 3**).

These results prompted to investigate in a pilot-experiment the effects in a syngeneic *in vivo* model (BALB/c mice), in which tumors were injected in the flanks of the mice. Mice were irradiated with 10 Gy and Cabozantinib was administered as described above. However, the results revealed no differences between the group administered with Cabozantinib only versus combination with 10 Gy x-rays with respect to tumor growth control and amount of superficial lung metastases (data not shown). To account for the differences for tumor cell inactivation *in vivo* as compared to our *in vitro* studies, we increased the dose given to tumors to 14 Gy in a further experiment.

**Figure 4:**
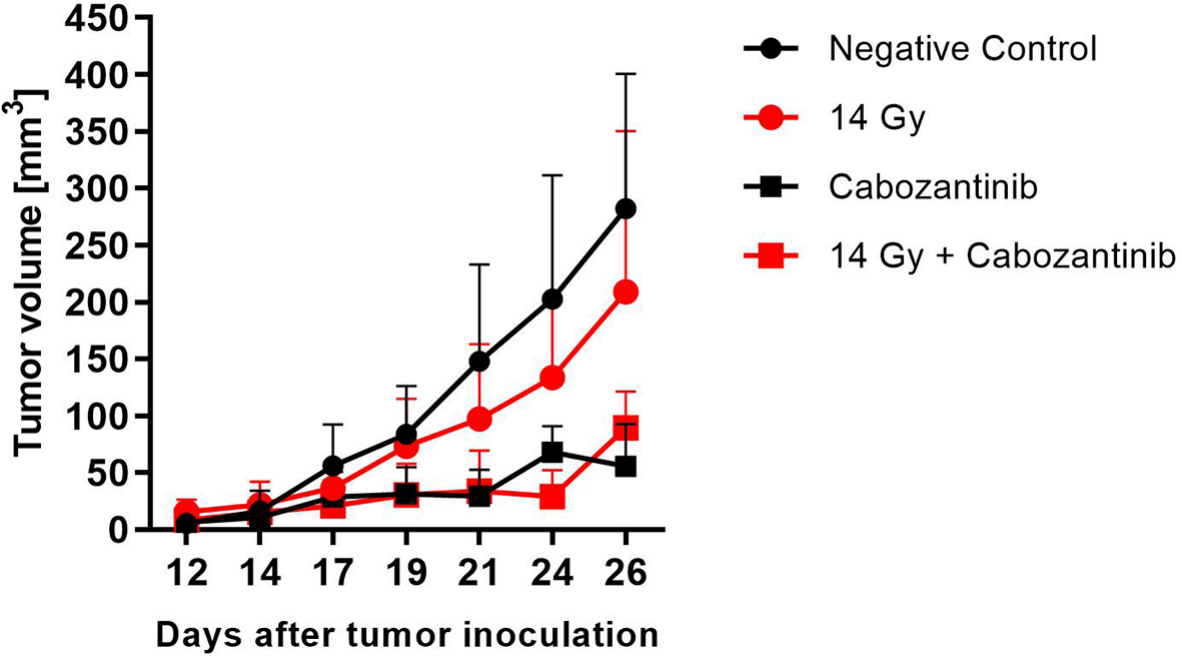
Tumor growth after treatment with x-rays and Cabozantinib. The tumor volume was measured with a caliper and the mean value (+SD, N=8-12) is plotted against days after tumor inoculation. Mice assigned to irradiation with 14 Gy of x-rays were exposed on day 14 and those assigned to Cabozantinib administration received treatment beginning on day 5. Significance was tested using a One-way ANOVA (p=0.068).

When tumors were irradiated with 14 Gy but mice were not treated with Cabozantinib, tumor growth was only slightly delayed and not significantly different from the untreated controls (**Figure 4**). In contrast, the administration of Cabozantinib to the mice starting from day 5 after tumor inoculation resulted in a significantly reduced tumor volume (p=0.002 and 0.006, Mann-Whitney U Test, without and with irradiation, respectively), as compared to the untreated controls. However, when the tumors of mice were treated with Cabozantinib and additionally irradiated with 14 Gy, no differences were found as compared to tumors in mice only administered with Cabozantinib. In summary, while Cabozantinib added on the effects of tumor cell inactivation *in vitro*, this was not reflected by the combined treatment *in vivo* with respect to tumor growth control, since the combined treatment regime did not show significant differences to the group administered with Cabozantinib only. In order to screen for systemic effects, we additionally investigated lungs and livers of the mice.

### Systemic Responses to the Combined Treatment Indicate a Reduced Recruitment of MDSCs to the Liver but No Reduction of Metastases in the Lung

**Figure 5:**
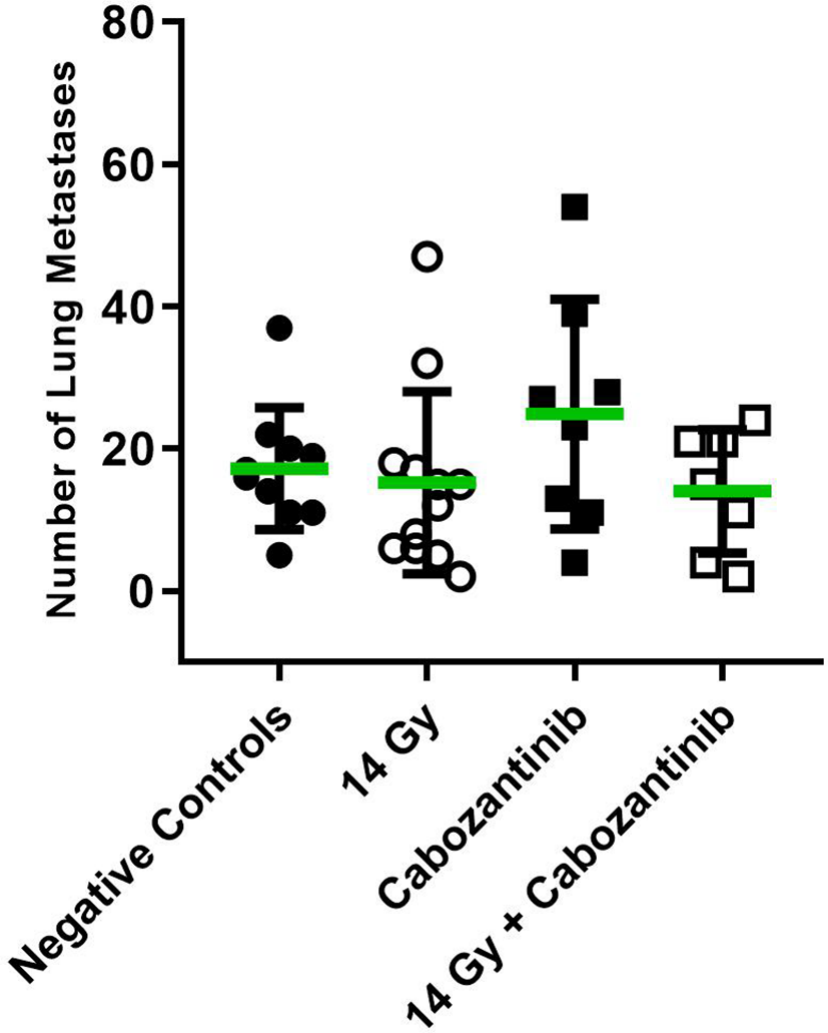
Number of superficially visible lung metastases following the different treatment regimens. Mice were treated with 14 Gy, Cabozantinib or both. Upon scarification on day 26 and resection, lungs were stained as described and the number of superficially visible metastases were counted (N=8-12). Significance was tested using a One-way ANOVA (p=0.2771).

**Figure 6:**
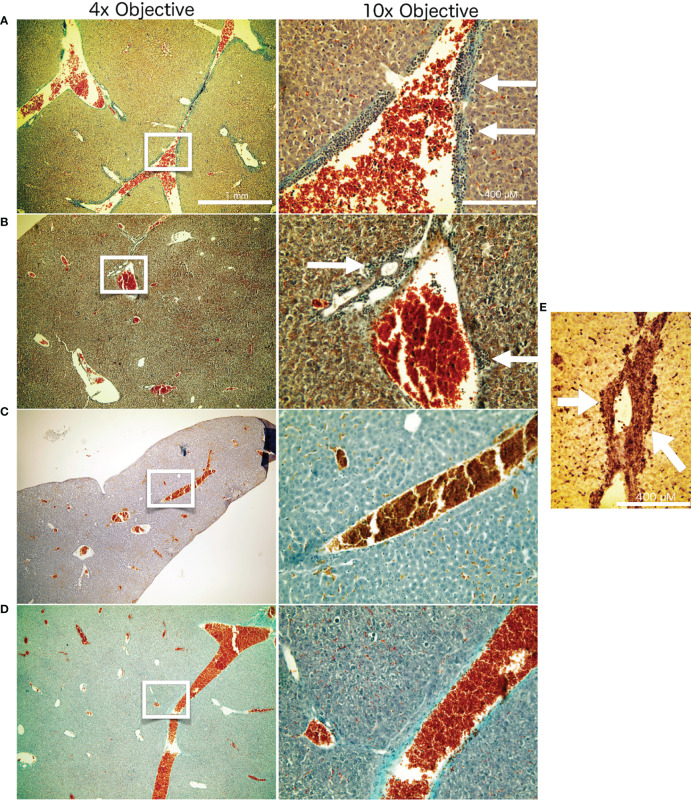
Liver histology. Masson Goldner staining of liver tissue from the different treatment groups after termination of the experiment at day 26. The white frames on the left column of images indicate the image seen in higher magnification in the right column. Represented are examples from the respective treatment groups: Untreated negative controls **(A)**. Mice which received 14 Gy of x-ray irradiation localized to the tumor at day 14 **(B)**. Mice which received Cabozantinib at 10 mg/kg per day beginning with day 5 after tumor inoculation **(C)**. Mice which received both 14 Gy irradiation to the tumor and the diet supplemented with Cabozantinib **(D)**. White arrows indicate the patches of CD11b-positive cells, indicating MDSC. **(E)** Representative CD11b staining of a liver sample from a untreated mouse 26 days after tumor inoculation. White arrows are pointing to the patches of CD11b-positive cells adjacent to liver vessels.

To investigate for the systemic anti-tumor immune response, we assessed the number of superficially visible metastases on the lungs of the animals upon staining. The results did not reveal significant reduction of the number of lung metastases as compared to the controls (**Figure 5**). While no macroscopically visible tumors were found in the livers of the animals of all groups, histology of the liver tissue of tumor bearing, untreated mice did show patches of cell infiltrates along the vessels, which were found to stain positive for the myeloid cell marker CD11b (**Figure 6**). The prevalence of such cells was also seen in livers from mice which received irradiation. However, in livers from mice which were treated with Cabozantinib with and without irradiation, this type of cells was reduced in prevalence or entirely absent. Hence, despite no effect on the reduction of metastases was found, the difference in recruitment of CD11b-positive cells, indicating MDSCs, points to a systemic effect.

**Figure 7:**
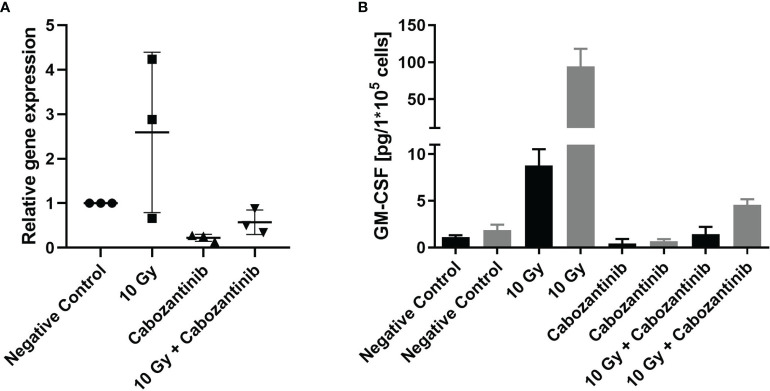
Gene expression and protein release of GM-CSF following treatment. Cells were treated as described with irradiation and/or Cabozantinib. Gene expression analysis 24h after treatment **(A)**. Data are derived from three independent experiments each and show the n-fold as compared to each negative control. Analysis with a One-way ANOVA revealed no significant differences between the groups (p=0.0537). Release of GM-CSF measured in the supernatant 24h (black columns) and 48h (grey columns) after treatment **(B)**. Release of GM-CSF is normalized to the cell number. Data are derived from one experiment carried out in triplicates, which were each measured in technical duplicates. Results were found to be significant following an analysis using a One-way ANOVA (p<0.0001, for 24h and 48h, both negative controls vs. 10 Gy and 10 Gy vs. 10 Gy + Cabozantinib).

To support these findings, we conducted *in vitro* studies assessing the expression and release of GM-CSF, which was reported to play a major role in the recruitment and the immune-suppressive capabilities of 4T1 cells (14). Irradiation with 10 Gy alone resulted in a pronounced increase of both expression and release (significant) of GM-CSF 24h upon irradiation as compared to negative controls (**Figure 7**). Instead, when cells were treated with Cabozantinib alone, expression and release were found to be reduced and the combination of both treatments resulted in a mitigation of the effects of irradiation alone.

## Discussion

Treatment of triple-negative breast cancer remains a challenge since it is highly invasive and features a low response to therapeutics, often leading to relapse (10). Combined therapies may offer vast potential in treatment of metastatic disease in general when systemic immune responses are triggered. Radiotherapy has been shown to trigger such effects and it is under discussion whether an SBRT-like dose administration may be particularly efficient (12). Likewise, Cabozantinib increased the number of circulating CD8+ T-cells and decreased the presence of CD14+ myeloid cells in a Phase II study (11). In an *in vivo* prostate cancer model, Cabozantinib was reported to eradicate advanced tumors by activation of anti-tumor immunity (15). We therefore hypothesized that a combination of RT in a SBRT-like character and Cabozantinib may be beneficial in the treatment of TNBC. We investigated the potential of such therapy using the murine 4T1 model of metastatic breast cancer.

A combination of Cabozantinib in 2.5 µM with 10 Gy of x-rays, both having demonstrated to cease the growth of 4T1 cells (**Figure 1**), revealed a slightly synergistic effect as compared to the single treatments when cell death was quantified (**Figure 3**). However, irradiation mainly contributed to the reduction of living cells in the cultures. Furthermore, the addition of Cabozantinib to irradiation with x-rays resulted in an RBE of about 2 with respect to clonogenic cell survival (**Figure 2**). Hence, our data suggested a direct effect of drug regimen and irradiation on 4T1 tumor cells. Given these promising results, we investigated the effects on local tumor control and systemic effects in lung and liver in a syngenic BALB/c mouse model.

Treatment with Cabozantinib beginning at day 5 after tumor inoculation throughout the remaining 21 days of the study resulted in a significant tumor growth reduction (p = 0.0002). This is in line with the reported suppression of tumor growth in 4T1 tumor models upon inhibition of VEGFR-2 and c-Met (16), which are kinase targets of Cabozantinib (17). However, x-ray irradiation at a dose of 14 Gy applied to the 4T1 tumors did not lead to a significant difference to the control group with respect to tumor growth. This points to a high radioresistance in our *in vivo* model, which is in contrast to former work reporting a significantly reduced tumor growth in a similar model system, already after 12 Gy exposure to the tumor (18).

Systemically, it was shown that irradiated 4T1 tumors can attract mesenchymal stem cells (MSC) to the tumor (19), which can give rise to immune suppressive fibroblasts, which attract MDSC to the tumor and raise other features of treatment resistance (20). In the work presented here, the combination of both treatment measures did not result in further reduction of tumor growth as compared to Cabozantinib alone (**Figure 4**), showing that synergistic effects as seen in the *in vitro* studies were not reflected *in vivo*.

We also aimed at investigating the potential of the combined treatment with Cabozantinib and irradiation with respect to systemic immune responses. For Cabozantinib, anti metastatic activity was reported also in immune incompetent tumor models due to the inhibitory effect on VEGF-R and c-Met (21). In our set-up, however, despite the reported anti metastatic activity of Cabozantinib (21), neither of the treatment methods nor the combination resulted in a reduction of lung metastases count (**Figure 5**).

Instead, an indication for a systemic immune-related response was found analyzing the livers of the animals. Liver histology of tumor bearing, untreated mice revealed cell infiltrates along the vessels, which were positive for the myeloid cell marker CD11b (**Figure 6**), indicating presence of MDSC (22). In the 4T1 and other tumor models, the patches of cells visible along the liver vessels have been characterized as an accumulation of immune suppressive MDSC (23). The expansion of the immune suppressive MDSC was shown to be possible with injection of GM-CSF in absence of a tumor, and adoptively transferred MDSC were also shown to home to the liver, where they closely resembled their presence in the spleen (23). In the 4T1 model specifically, the immune suppressive capability of these Cd11b^+^/Gr-1^int^ MDSC was shown to be steered exclusively by GM-CSF (14). Under influence of Cabozantinib, there was no visible presence of these cells in the liver. This is in line with the reported activity of Cabozantinib in antagonizing MDSC presence and with our results for gene expression and relsease of GM-CSF, which did show a reduced expression and protein presence of GM-CSF in 4T1 cells and in their supernatant, respectively (**Figure 7**).

Here, the prevalence of such cells was observed in livers from mice that received irradiation. However, in livers from mice that were treated with Cabozantinib with and without irradiation, this type of cells was reduced in prevalence or entirely absent, in line with the capability of Cabozantinib to antagonize MDSC as shown in other models (7, 8). Moreover, enhanced release of GM-CSF following irradiation of 4T1 tumors was shown to attract circulating tumor cells into the tumor bed of irradiated tumors and unirradiated secondary tumor sites, contributing to tumor re-growth (24). Cabozantinib, on the other hand, resulted in a reduced tumor growth (**Figure 4**), thus pointing to a role for GM-CSF and MDSC recruitment in our model and an explanation for the radioresistance of tumors treated with irradiation alone.

In conclusion, despite promising synergistic *in vitro* effects, the combination of photon radiotherapy and Cabozantinib failed to add on the effect of Cabozantinib alone with respect to tumor growth control. Furthermore, none of the treatments, in combination or alone revealed anti-metastatic effects. However, the ability of Cabozantinib to antagonize an increase in GM-CSF in 4T1 cells following irradiation indicates a utility in combination with radiotherapy, despite the lack of synergistic effects in terms of tumor growth or metastases control in our study.

## Data Availability Statement

The raw data supporting the conclusions of this article will be made available by the authors, without undue reservation.

## Ethics Statement

The corresponding ethical approval code is 23 177-07/G 15-8-058.

## Author Contributions

NR, AH, and LD have substantially contributed to the conception and design of the work, as well as the acquisition, analysis and interpretation of the data. AH, MD, and CF have substantially contributed to design and revision of the manuscript. All authors contributed to the article and approved the submitted version.

## Funding

This work was partially supported by ESA-IBER project and FAIR-phase-0.

## Conflict of Interest

The authors declare that the research was conducted in the absence of any commercial or financial relationships that could be construed as a potential conflict of interest.

## Publisher’s Note

All claims expressed in this article are solely those of the authors and do not necessarily represent those of their affiliated organizations, or those of the publisher, the editors and the reviewers. Any product that may be evaluated in this article, or claim that may be made by its manufacturer, is not guaranteed or endorsed by the publisher.
