# Supplementary material for: A Combination of Cabozantinib and Radiation Does Not Lead to an Improved Growth Control of Tumors in a Preclinical 4T1 Breast Cancer Model
Source: Front Oncol. 2021 Dec 8;11:788182. doi: 10.3389/fonc.2021.788182 (PMC8692262; doi:10.3389/fonc.2021.788182)
Supplement: Supplementary file 1 [file Table_1.docx]

**Supplementary material**

Table 1. **Parameters resulting from linear-quadratic fits of the data stemming from clonogenic cell survival (Fig. 2).** A Student’s t-test revealed significant differences for α (p<0.001) but nor for β (p>0.1).

|  | **α** | **β** | **α/β** | **σ(α)** | **σ(β)** |
| --- | --- | --- | --- | --- | --- |
| x-rays | 0.0783 | 0.0571 | 1.371 | -0.0121 to 0.0408 | -0.0123 to 0.0173 |
| x-rays+Cabozantinib | 0.4831 | 0.0654 | 7.392 | 0.0314 to 0.2408 | -0.0034 to 0.0293 |
